# Life History, Identity, and Recovery in People with Mental Health Conditions: A Phenomenological Study Using OPHI-II

**DOI:** 10.3390/healthcare14010003

**Published:** 2025-12-19

**Authors:** Olga I. Fernández-Rodríguez, Alicia Cal-Herrera, María Fernández-Blanco, Paloma Guillén-Rogel, Beatriz Fernández-Díez, Raquel Martínez-Sinovas

**Affiliations:** INPRO Research Group, European University Miguel de Cervantes, 47012 Valladolid, Spain; oifernandez@uemc.es (O.I.F.-R.);

**Keywords:** mental health, narration, Occupational Therapy, patient-centered care, quality of life, social support

## Abstract

**Background and Objectives**: Mental health recovery is conceived as a personal process going beyond symptom remission and thus involving identity reconstruction, search for meaning and active participation in everyday life. This study aimed to analyze the influence of life history, identity, competence and context on the recovery process of people with mental illness. **Methods**: A qualitative phenomenological study was conducted and registered in the Open Science Framework. Participants were 159 individuals diagnosed with mental disorders who attended a community mental health association. Data were collected using the standardized Occupational Performance History Interview-II (OPHI-II) and analyzed through an inductive phenomenological approach with researcher triangulation. **Results**: Findings showed that life history is central to recovery, as it helps rebuild identity, recognize personal capacities and restore a sense of continuity. Daily occupations and social support emerged as key factors for inclusion and participation. **Conclusions**: Results highlight the importance of integrating biographical narratives and occupational perspective into mental health interventions through a standardized tool that surpasses traditional diagnosis-based or methodologically weak approaches. This perspective fosters practices aligned with individuals’ values, goals and contexts, promoting autonomy, empowerment, and social inclusion. These findings may inform person-centered recovery programs in community services.

## 1. Introduction

Mental health is a dynamic and complex process ranging from psychological well-being to the most debilitating states of emotional suffering [1]. The World Health Organization World Report on Mental Health estimates that more than one billion people, one in eight, suffer from some form of mental disorder, with depression (280 million cases) and anxiety disorders (301 million) being the most prevalent [1]. In Spain, around 34% of the population reports having experienced some kind of mental health problem, with sleep disorders, anxiety and depression being particularly prevalent [2].

The chronicity of mental disorders is one of the greatest challenges for healthcare systems due to frequent relapses, functional loss and high socioeconomic costs, with added factors such as late detection, treatment discontinuity, and social stigmatization that further hinder sustained recovery [3]. Although deinstitutionalization has promoted community inclusion, it has also highlighted limitations in support resources and psychosocial rehabilitation programs [4].

Given this situation, there is a need to design comprehensive community programs that address the complexity of mental disorders and promote recovery that goes beyond mere symptom remission. This process involves rebuilding identity, strengthening the sense of belonging and fostering personal empowerment, which are fundamental principles of the person-centered recovery model [5]. Furthermore, all of these therapeutic goals are based on hope, collaboration, development of personal strengths, supporting self-determination, self-care and social inclusion [6], which closely aligns with the CHIME model and its five essential components of personal recovery: connection, hope, identity, meaning and empowerment, as pillars of a meaningful and socially integrated life [7].

The active participation of individuals in decision-making regarding their treatment is key to strengthening adherence and commitment to their own process of recovery [8]. From this perspective, interventions should be aimed at supporting the reconstruction of each individual’s life project, providing the necessary resources for them to achieve their personal goals. Moreover, understanding this life project requires exploring the person’s life history, identifying the significant events that have shaped their identity and performance in specific social and physical contexts.

In addition, the Human Occupation Model (MOHO) provides a theoretical framework for analyzing how personal factors, volition, habituation, and performance capacity interact with the environment to shape personal and occupational identity [9]. Both the CHIME framework and MOHO offer complementary theoretical perspectives that enrich the understanding of mental health recovery, particularly in relation to identity development, volition and participation in meaningful daily activities. The CHIME framework domains emphasize the reconstruction of a coherent sense of self, the restoration of agency, and the cultivation of meaningful roles, elements that are central to navigating life with a mental health condition. Similarly, MOHO provides an occupation-centered lens through which recovery can be examined by focusing on volition, habituation, performance capacity and the environment. Identity and wellbeing are shaped through engagement in personally meaningful occupations, the development of roles and routines, and the interaction between individuals and their social and physical contexts [10]. Integrating CHIME and MOHO offers a robust conceptual foundation for understanding how individuals rebuild their lives amidst mental health challenges.

In this context, the Occupational Performance History Interview–II (OPHI-II) enables an in-depth narrative exploration of individuals’ life trajectories, shedding light on the meaning people ascribe to their occupations and to their recovery process. Its use has proven useful in identifying meaningful roles, values and routines that guide person-centered therapeutic planning [11,12]. In addition, various studies confirm its validity and adaptability in different clinical and community contexts [13,14,15,16,17,18,19,20,21].

However, most previous studies have focused on the psychometric aspects or technical application of the OPHI-II, without delving into how people experience and attribute meaning to their life story in the recovery process. Similarly, existing research using comparable conceptual frameworks tend to rely exclusively on the CHIME model, involve samples composed mainly of women with mood disorders, is conducted in inpatient or hospital-based settings, or does not employ standardized tools for identity assessment [22,23,24,25]. This gap in the literature underscores the need for qualitative approaches that can capture the subjective experience of individuals living with mental disorders from their own perspective, framed within the CHIME and MOHO models.

Thus, this study adopts a phenomenological approach, aimed at exploring in depth how life history, identity, competence and context influence the recovery process of people with mental health problems. This approach allows us to recognize the uniqueness of each life trajectory and provide qualitative evidence to strengthen person-centered and recovery-oriented interventions.

## 2. Materials and Methods

### 2.1. Study Design

This qualitative study is part of a mixed-methods research project registered on the Open Science Framework (OSF; https://doi.org/10.17605/OSF.IO/6BVKD). A qualitative design with a phenomenological approach was developed, which is ideal for exploring and gaining an in-depth understanding of the experiences. Phenomenology is a qualitative research approach that focuses on exploring and understanding individuals lived experiences and the meanings they ascribe to them. It is based on the principle that human experiences can be examined to reveal their essential structures, allowing researchers to gain in-depth insights into subjective perspectives and the ways people perceive and make sense of their realities [26]. This approach offers insight into how participants make sense of their life history, identity, competence and context, all of which are key components for the recovery process for people with mental illness.

The main objective was to analyze how these factors influence the construction of the recovery experience from the perspective of the participants themselves. The study is reported in accordance with the COREQ (Consolidated Criteria for Reporting Qualitative Research) guidelines, ensuring transparency, consistency and methodological rigor in all phases of the research process.

### 2.2. Participants and Sampling

The qualitative study was conducted with the same 159 participants included in the quantitative phase of the mixed-methods project, as the aim was to gain a comprehensive understanding of the recovery process by combining objective and narrative information. Participants were selected using purposive sampling, which allowed for the inclusion of individuals with experiences directly related to the phenomenon under study.

The inclusion criteria were: (1) being over 18 years of age, (2) having a mental illness diagnosis, (3) requiring daily life support and (4) agreeing to voluntarily participate. Exclusion criteria were: (1) substance use and (2) active psychotic symptoms that interfered with understanding the interview (e.g., hallucinations, delusions, or disorganized thinking).

The sample size was justified according to the principle of information power, which considers the adequacy of the participants number based on the relevance and depth of the data, the sample specificity and the study objectives. Given the clearly defined approach, the intentional selection of participants and the richness of the narratives obtained, it was considered that the inclusion of 159 interviews provided sufficient information power for phenomenological analysis. The decision to rely on this approach rather than data saturation aligns with contemporary methodological recommendations, as information power is considered more suitable for phenomenological designs that prioritize depth, relevance and nuance over thematic repetition [27].

Participants were recruited through *El Puente Salud Mental* community association (Valladolid, Spain). All received detailed information about the objectives, procedures, benefits and possible study risks, ensuring an informed and voluntary decision.

### 2.3. Community Mental Health Resource and Procedures

As mentioned, the study was conducted in collaboration with *El Puente Salud Mental*, a non-profit community organization that provides psychosocial support to individuals experiencing mental health conditions. Its mission is centered on promoting social, occupational and community inclusion through a range of interventions aimed at enhancing personal autonomy, supporting life-project development, fostering socio-vocational integration and facilitating participation in leisure and community activities.

The service operates under a model of comprehensive accompaniment, offering flexible scheduling and designated reference professionals for each service user. Activities are delivered through structured programs, which allows for consistent support but also introduces some constraints regarding the personal choice for activities.

Interviews were conducted in person at the association’s headquarters and lasted approximately 60 to 90 min. They were conducted by the principal investigator and another occupational therapist with experience in mental health, together with a fourth-year student studying for an Occupational Therapy degree under direct supervision of the principal investigator. All interviews were scheduled within flexible time slots selected by participants, ensuring comfort, accessibility and minimal disruption to their daily routines. This procedure ensured consistency in the instrument application as well as in data collection. Furthermore, the interviewing team was not part of the association.

### 2.4. Data Collection

Data collection took place between April and May 2024. The OPHI-II tool was used. This tool was designed to explore the person’s occupational history through a semi-structured interview, in order to analyze how identity, competence and context influence their life trajectory. Evidence of the OPHI-II’s validity in mental health populations, along with its broad use in community settings, supports its relevance and suitability for this research [11].

For ethical reasons and to avoid the use of tape recorders reducing the participants’ willingness to collaborate, it was decided not to record the interviews. Considering the characteristics of our sample, we believed that the presence of recording devices could exacerbate positive symptomatology among individuals with severe mental health conditions, potentially generating distress or leading many participants to refuse involvement altogether. Therefore, they were transcribed verbatim during the interview. To reinforce reliability, complementary strategies were applied, such as cross-checking the transcripts by more than one researcher, triangulation during analysis and the use of field notes, in which interviewers recorded relevant contextual information and interpretive notes immediately after each session.

### 2.5. Data Analysis

The information was analyzed using thematic analysis, following the procedure described by Braun and Clarke, comprising six phases: (1) familiarization with data, (2) generation of initial codes, (3) search for themes, (4) themes review, (5) themes definition and naming and (6) final report preparation [28]. The process was carried out by an external team specializing in qualitative methods with accredited experience in qualitative methodology and data analysis in the health sciences. These experts were selected through targeted invitation, based on their academic trajectory and recent publications in qualitative research, and had no affiliation with the data collection context.

To ensure the coherence and credibility of the analysis, regular meetings were held with the principal research team, during which the codes, categories and emerging themes were critically reviewed and discussed until consensus was reached. This external triangulation strategy helped to minimize potential individual biases and to strengthen the validity and interpretive robustness of the findings. ATLAS.ti software (25 version, ATLAS.ti Scientific Software Development GmbH, Berlin, Germany) was used to organize, code and manage data, as well as to maintain a systematic record of analytical decisions.

### 2.6. Ethical Considerations

The study was approved by the Ethics Committee of the first author’s institution (code: 14/2023). The principles of the Declaration of Helsinki (Fortaleza, 2013) and current Spanish legislation (Law 14/2007 and Royal Decree 223/2004) were respected).

All participants or their legal representatives where applicable, signed a written informed consent form before participating. Confidentiality and anonymity were guaranteed through the use of code instead of personal data.

### 2.7. Methodological Rigor and Quality

To ensure the methodological quality of the study, various strategies were applied in accordance with credibility, reliability, confirmability and transferability criteria.

Credibility was strengthened through both the triangulation of researchers and the participation of an external team of university experts specializing in qualitative analysis, who provided an independent perspective and reduced possible biases arising from the proximity of the field team investigators to participants. As mentioned, regular meetings were held to discuss codes, categories and emerging themes until interpretive consensus was reached.

Dependability was ensured through the systematic use of ATLAS.ti software, allowing detailed recording of analytical decisions and ensuring coding and categorization process traceability.

Confirmability was reinforced through transparent recording of analytical decisions and inter-researcher validation, ensuring that findings were based on data rather than individual interpretations. During the study, researchers kept a reflective journal to record perceptions, expectations and possible personal influences throughout fieldwork and analysis. This procedure promoted transparency, self-criticism and adherence to COREQ criteria, reinforcing the interpretive consistency of the study.

Transferability was promoted through detailed participants’ descriptions, selection criteria and data collection context. Thus, results were accompanied by representative quotes that allow the reader to assess the findings’ applicability in other settings or populations.

Interviews were conducted by occupational therapists with no prior relationship or professional connection to participants, minimizing potential familiarity or hierarchy biases. Information power was considered an interpretive rather than a statistical criterion, based on the narratives’ richness and depth in relation to the study objective.

## 3. Results

A total of 159 adults diagnosed with mental illness were recruited, all of whom were in the process of recovery. Of these, 108 were men (67.9%) and 51 were women (32.1%). The mean age of the sample was 49 years old (SD 12.47).

Regarding mental illness diagnoses, although the same person may present up to three different pathologies, it was found that 41.5% of participants were classified within the schizophrenia spectrum disorder, 26.4% with depressive and bipolar disorders and 20.1% with personality disorders.

After performing the thematic coding analysis, four themes were identified: (a) daily routine: between stability and monotony; (b) limited occupational roles and performance; (c) restricted support and feelings of loneliness; (d) goals, projects and perception of the future. Analysis of the interviews allowed us to identify common and unique experiences related to the participants’ occupational performance, social participation and sense of purpose.

Results are presented with narratives examples obtained from participant interviews. The use of narratives helps maintain the results’ credibility and traceability [29].

### 3.1. Theme 1: Daily Routine: Between Stability and Monotony

#### 3.1.1. Subtheme 1.1.: Structured Daily Routines

Participants describe daily life organized around repetitive and predictable activities. In most cases, routines consist of three main lines:

Workshops attendance: they describe community spaces offering a certain daily structure, where interviewees often do crafts, occupational tasks, or simply socialize with others. These workshops are described as an important framework for maintaining a certain routine regularity: “I go to the workshop, then I come home, clean a little and then I turn the TV on” (P36).

Household chores: cleaning, cooking and home maintenance appear to be activities carrying a lot of weight in daily routine. Some participants report engaging in these occupations as a way to feel useful and to exert control over their immediate surroundings. “What I like most is hanging laundry; it keeps me entertained” (P105).

Passive leisure: much of their free time is spent on solitary, low-demand activities such as watching television, listening to music, or taking short walks: “when I get home, I turn the TV on and that’s how I spend my day” (P36).

Although these routines show organization and provide daily life stability, their lack of variety is described by participants as limiting opportunities to explore more meaningful or socially integrating activities.

#### 3.1.2. Subtheme 1.2.: Perception of Routine

The subjective experience of daily routines is ambivalent. Some participants positively describe the predictability and tranquility or calm that the repetition of activities brings them, saying that it helps them to remain stable and feel secure. They sometimes perceive routine as a “refuge” that brings them peace of mind. “I am satisfied with my current routine; I prefer to be calm” (P95).

On the other hand, a group of participants describe dissatisfaction and boredom, associating routine with monotony, lack of motivation, and/or absence of a purpose in life. These perceptions are often accompanied by feelings of emptiness or stagnation. “My life is monotonous and I lack purpose” (P2). “I always do the same thing, every day is the same” (P54).

This duality reflects that routine assessment depends on the meaning that each person gives it, their level of involvement and the possibilities of choosing meaningful activities in line with their interests: “what I like most is hanging out the washing” (P105). For some, it represents a source of stability, while for others it becomes an obstacle to advancing their personal projects.

### 3.2. Theme 2: Roles and Limited Occupational Performance

#### 3.2.1. Subtheme 2.1.: Predominant Roles

Participants show how the most frequent occupational roles are linked to home and community life. Many take on domestic responsibilities such as cleaning, cooking, or shopping, activities that sometimes become the main source of daily structure: “I am proud to clean the garden” (P55). In addition, several interviewees participate in occupational or community workshops, which serve as spaces for socialization and learning. “I go to the workshop almost every day, so I have something to do” (P36).

Likewise, the caregiver role appears in some cases, especially in relation to older family members or those in need of support: “taking care of my father is what gives me strength every day” (P21). Although this role is valued as significant, it can also be experienced as a burden that limits the development of other personal interests. “my emotional independence is limited because I depend on them, but at the same time they depend on me” (P51).

These narratives show how, even in the absence of formal employment, people draw on domestic and community roles as a way to preserve their occupational identity and feel useful in their immediate environment.

#### 3.2.2. Subtheme 2.2.: Limitations in the Work Role

Most participants point out that their work trajectories have been interrupted due to mental health issues, physical disabilities, or critical situations such as accidents or prolonged hospitalizations: “my life changed after the motorcycle accident. I lost my job and all motivation” (P92). This loss of the work role generates feelings of frustration and, in some cases, personal devaluation: “when I was working, I felt independent, now I depend on others for everything” (P27) or “since I stopped working, I feel like I’m no longer good for anything” (P45) are examples that show these emotional states.

The inability to re-enter the formal labor market not only impacts personal finances, but the perception of autonomy and social recognition as well. “I stopped working when I had a relapse and never went back” (P44).

Despite these limitations, some participants express a desire to return to work, although they recognize the existence of multiple barriers: “I would like to work, but I no longer feel capable” (P33). This desire coexists with a resigned acceptance that, in their current situation, possibilities are slim. “I would like to go back to work, even if it’s only for a few hours, to feel useful again” (P33). “I know I’m no longer in a position to do so, but I still dream of having a simple job” (P61).

Moreover, participants report that the absence of a work role is not just a matter of employment, but a factor directly affecting identity, self-esteem and the ability to plan for the future.

#### 3.2.3. Subtheme 2.3.: Achievements and the Feeling of Self-Efficacy

Many participants emphasize the importance of recognizing and valuing small achievements in their daily lives, which are described as milestones that strengthen self-esteem and generate a sense of self-efficacy.

For example, simple domestic activities, such as cleaning, tidying up, or cooking, are referred to with pride since they allow them to experience control over their immediate environment. “I feel proud of cleaning the garden because it’s something I do on my own and it looks nice” (P55), “what I like most is hanging out the washing because I know I’ve done it myself and it looks good” (P105), “tidying my room calms me down, it gives me peace of mind to know that everything is in its place” (P71). These statements reflect how routine tasks take on symbolic value when perceived as expressions of autonomy and ability: “when I mop the house, I feel useful, like I can contribute something” (P38).

Participation in community workshops is referred to as an opportunity for learning and personal validation: “when I do crafts in the workshop, I feel like I can do something well” (P14), “going to the workshop cheers me up, it gives me the strength to leave the house” (P36). For some participants, these experiences represent a way to rebuild their occupational identity after losing formal employment: “I used to work in an office and I miss it, but now in the workshop I feel part of something, I feel valued” (P62).

These achievements perceived by the participants, although often small and linked to the domestic or community sphere, become motivation and resilience drivers. Through them, participants perceive progress in their performance, reinforcing confidence in their abilities and creating the possibility of setting new goals: “tidying my room calms me down, gives me peace of mind, and then I think I could also tidy up other things in my life” (P71).

However, several participants also express the need for external recognition or validation. For some, the approval of family members or professionals is crucial to giving true value to their achievements and improving their sense of self-efficacy: “I like it when they tell me I did well, it encourages me to keep going” (P41). In addition, some participants point out that workshops are a place for socialization and mutual support. Thus, they are described as a protective environment that not only promotes their individual achievements but is also linked to social participation: “I talk to people there, I feel accompanied” (P63).

### 3.3. Theme 3: Limited Support and Feelings of Loneliness

#### 3.3.1. Subtheme 3.1.: Family Support

Family appears to be the main source of support in many participants’ lives, both emotionally and practically. Several participants report their family members are their most important source of trust: “I trust my brother and my cat. They are the most important thing” (P95). This support provides an indispensable foundation of security and companionship for day-to-day life, as showed on the following quote “if it weren’t for my mother, I don’t know how I would get by” (P21).

However, this dependence also creates tensions within the family. In some cases, participants describe how their relationship with their family members limits their autonomy: “my emotional independence is limited because I depend on them” (P51). In other cases, family dynamics are referred to as a source of conflict, especially when differences arise around the organization of daily life or the degree of supervision exercised by family members. These tensions generate feelings of discomfort and lack of control. “I would like to decide more things for myself, but at home they always make decisions for me” (P37).

This duality describes how family can be seen, at the same time, as a source of resilience and as an obstacle to the development of the participants’ personal independence.

#### 3.3.2. Subtheme 3.2.: Social Relationships Outside the Family

Participants report that social interactions beyond the family nucleus are often limited to housemates or workshop colleagues. Some participants value these bonds as spaces for companionship and belonging, while others express a strong sense of isolation: “I feel lonely, I don’t have many friends, I have fun at the workshops, but outside of that, no one calls me” (P159), “I get along with my housemates, we chat and that’s enough for me” (P44).

This contrast reflects a tension between their need for companionship and the difficulties in establishing and maintaining social relationships beyond these core groups. While some find sufficient support networks in workshops or small social groups, others experience frustration at not being able to expand their ties to the wider community: “in the workshop, I talk to people and feel accompanied, but when I go home, I am alone, I have no one to go out with” (P63).

The difficulty in establishing new relationships is associated with mistrust of others and experiences of stigmatization that have reinforced social withdrawal: “I don’t hang out with people much because they look strangely at me” (P28).

#### 3.3.3. Subtheme 3.3.: Barriers to Community Integration

Beyond family and workshops, community participation is limited. The main barriers include a lack of accessible opportunities, the stigma associated with mental illness, and a perception of insecurity when interacting with strangers: “I find it hard to go out because I feel like I don’t fit in” (P63). Participants recognize the importance of feeling part of the community, yet most describe multiple obstacles that hinder their full integration. These barriers encompass personal, social, and structural factors that collectively reinforce the feeling of exclusion.

The stigma associated with mental illness appears to be one of the most significant barriers. Some describe experiences of rejection or discrimination that led them to limit their social participation to small circles. With examples from quotes such as “when they found out I had been admitted to a psychiatric ward, they started treating me differently, as if I were dangerous” (P29), “I stopped going out with my friends because I found out they were talking about me behind my back, saying I was crazy” (P47) or “once in the neighborhood, they yelled at me that I was sick and to go away. I hardly ever go out since then” (P34).

This situation underscores a marked inequality in access to meaningful social activities, heightening individuals’ vulnerability to isolation and limiting their opportunities for personal development. Several participants point out that, beyond workshops, they find few options suiting their needs: “outside the workshop, I don’t know where I could go; there are no places for us” (P46). This lack of alternatives limits socialization and recreation opportunities, reinforcing dependence on institutional spaces.

### 3.4. Theme 4: Goals, Projects, and Perception of the Future

#### 3.4.1. Subtheme 4.1.: Objectives and Goals for the Future

Some participants express clear and specific goals related to education and work, which become motivators and a way to envision a different future. These aspirations are often linked to a desire for self-improvement and the wish to regain a certain degree of autonomy. “I want to finish secondary school and study history, because I’ve always liked it and I want to prove to myself that I can still learn” (P5); “my goal is to set up a rural guest house” (P96). On several occasions, participants refer to academic training as an opportunity for growth and broadening their horizons. They associate these types of goals not only with the ability to acquire knowledge, but also with the ability to regain confidence and demonstrate their self-improvement capacity. Statements such as “when I think about going back to school, I feel like it would be starting over, like giving myself a second chance” (P18) or “I would like to take a computer course so I could find a job and prove that I am capable of supporting myself” (P33) show this connection.

Some participants refer to goals in the workplace as an opportunity for autonomy. Although they recognize the difficulties, this goal becomes a horizon that gives meaning to their daily efforts. Sometimes they express achievable short-term goals, such as finding a simple or part-time job: “I would like to go back to work, even if it’s only for a few hours, to feel useful again” (P33). Participants point to a link between work and personal identity. For some, work represents more than just earning money, it is also a way to reclaim a place in society and enhance self-esteem: “I want to have a simple job, even if I don’t earn much, just to prove that I can do it” (P61).

These goals are described by participants as a source of hope, helping to maintain their belief in the possibility of a different future. They function as anchors that organize motivation and allow them to maintain a sense of purpose, beyond their current difficulties.

Some participants describe broader, more subjective aspirations linked to the search for well-being, tranquility, and satisfaction in everyday life. These are not always clearly defined goals, but they express a desire to improve their quality of life and achieve a more stable emotional state: “my goal is to feel comfortable” (P100), “I would like to do more things outside the home, go out more” (P72).

These aspirations, although less concrete than academic or work-related ones, reflect the need to find balance, personal satisfaction, and a life less marked by monotony. For many participants, achieving emotional and social well-being is a meaningful goal in itself.

#### 3.4.2. Subtheme 4.2.: Obstacles to Achieving Goals

Despite their desires and future plans, participants describe numerous barriers that hinder their goals’ achievement. These include physical and cognitive limitations, lack of financial resources and emotional difficulties stemming from mental illness.

Physical, cognitive and emotional conditions are identified as determining factors in the difficulty of moving forward. Some participants acknowledge that, although they still want to work or study, their current condition limits their possibilities: “I know I’m no longer in a position to do so, but I still dream of having a simple job” (P61). The symptoms associated with mental illness, fatigue and lack of concentration emerge as daily obstacles interfering with perseverance and their ability to sustain long-term projects: “I want to do things, but depression stops me, I lose interest in everything and give up” (P73), “I have days when my head doesn’t work, I can’t think clearly, and so it’s impossible to continue with a project” (P67).

Another recurring aspect is the difficulty in maintaining effort over time. Several participants describe how they start out enthusiastically but quickly lose motivation: “I start studying with enthusiasm, but I get distracted right away, I can’t concentrate, and I give up” (P49). This discontinuity weakens the goal-setting process and reinforces feelings of ineffectiveness.

The lack of material resources is also perceived as a major obstacle to achieving goals. For some participants, economic problems make it unfeasible to access courses, recreational activities, or even more independent living conditions: “I want to become independent, have my own apartment, but it’s impossible with my earnings” (P41). This limitation reinforces dependence on family or institutional support. Likewise, social stigma and the scarcity of accessible job opportunities appear as structural obstacles: “I want to work, but I know they won’t hire me because when they see my medical history they don’t call me back, so I settle for workshops” (P36). This perception of exclusion from the labor market reinforces resignation, limiting hope for real change, with people expressing statements that show this mindset: “I went to interviews, but as soon as I say I have a mental disability, they tell me they’ll call me and they never do” (P58) and “I don’t even try anymore, because I know they’re going to say no; that’s why I settle for workshops” (P82).

## 4. Discussion

The findings of this study revealed that recovery processes in people with mental illness are deeply influenced by life history, identity, competence and context. The sample included in this study was predominantly male with a mean age of 49 years old and a prevalence for psychotic disorders, which is usually linked with these types of disorders [30].

Four central themes emerged from the phenomenological analysis: daily routine between stability and monotony; limited occupational roles and performance; restricted support and feelings of loneliness; and goals, projects and perceptions of the future. These themes, which are intrinsically grounded in the MOHO, offer a comprehensive view of how people rebuild their sense of identity and purpose amid the limitations imposed by mental illness.

Building on the insights generated by this study, it is essential to position our results within the broader literature on identity, recovery, and occupational meaning. Most existing research using comparable conceptual frameworks tends to rely exclusively on the CHIME model, involve samples composed mainly of women with mood disorders, is conducted in inpatient or hospital-based settings, or does not employ standardized tools for identity assessment [22,23,24,25].

In our study, in relation to daily life, participants described routines organized around predictable and repetitive activities, mainly centered on occupational workshops, domestic tasks and forms of passive leisure. This structure, while offering a sense of stability, was also perceived as a source of monotony and demotivation. The predictability of daily life generated, in many cases, feelings of emptiness and stagnation, while it provided tranquility and control over the environment in others. This finding partially coincided with previous research pointing to the duality of structured routines as mechanisms of both containment and restriction in psychosocial rehabilitation processes [24,31].

The analysis showed that participants linked most of their occupational roles to household activities or institutional spaces, reflecting the limited diversity of meaningful experiences available. The interruption of their work role, whether due to illness, physical disability, or critical episodes such as prolonged hospitalizations, was associated with feelings of frustration and loss of identity. The absence of employment was perceived not only as a lack of economic activity, but also as the loss of a place for recognition and self-esteem. These findings were consistent with studies that highlight the importance of work as a source of identity and sense of belonging in the recovery of people with mental disorders [32,33,34].

Thus, participants value small daily achievements, such as completing household chores or actively participating in workshops, as milestones that strengthen self-esteem and self-efficacy. These experiences were interpreted as moments of reconstruction of personal and occupational meaning after the loss of previous roles. Similarly, previous research has identified that recognition of everyday achievements promotes self-esteem and a sense of self-efficacy. It also contributes to strengthening the recovery process [22,23,25,35,36].

Family was identified as the main source of both emotional and practical support, yet its role was ambivalent. While it offered support and promoted resilience, it could also create tensions due to overprotection or constrained autonomy. This ambivalence is consistent with previous research, which recognizes family both as an essential resource and as a factor that can restrict independence and perpetuate dependence dynamics [37,38,39].

In terms of social relationships, participants noted that their network of interaction outside the family nucleus is limited, restricted almost exclusively to housemates or workshop colleagues. While some valued these ties as opportunities for companionship, others expressed feelings of isolation and social withdrawal. These experiences reflect the impact of stigma and mistrust of others, which is consistent with studies that have documented the persistence of social barriers and discrimination against people with mental illness [40,41].

Community participation was described as limited, a factor influenced by limited access to opportunities as well as feelings of insecurity and internalized stigma. This set of structural and social barriers reinforces exclusion and restricts opportunities for meaningful participation. These findings complement existing evidence on the influence of sociocultural context on inclusion and recovery processes [42,43,44,45].

Finally, the participants’ narratives revealed the presence of goals and aspirations, both concrete (related to education or work) and more subjective (related to well-being or peace of mind). These goals were perceived as anchors sustaining hope and motivation, even in the face of personal and structural limitations. However, difficulties in maintaining long-term effort, lack of resources and employment stigma were identified as persistent obstacles. These results matched studies pointing to the importance of personal goals as a driver of the recovery process, as well as the need for environments that facilitate their achievement [46,47].

Overall, these findings suggest that recovery is not a linear or exclusively individual process, but rather an interdependent experience where routines, roles, support, and goals are intertwined with contextual and social factors. This understanding lays the foundation for deeper reflection on the meanings people attribute to their daily lives and how these findings dialogue with existing literature on identity, participation and inclusion in mental health.

From a phenomenological interpretation, recovery was understood as a dynamic process of reconstructing one’s sense of self, in which people sought to reestablish coherence between their past biography, their current conditions and their future aspirations. Mental illness was experienced as a rupture in the continuity of their self, forcing a redefinition of identity and way of being in the world. In this context, routines emerged as scenarios where individuals negotiated their sense of normality: when activities were meaningful, they offered a path to stability and integration; when they lacked purpose, they intensified the feeling of emptiness. In this way, daily life became an existential space where people attempted to rebuild their identity, rather than a mere sequence of functional acts. Phenomenology thus allowed everyday action to be interpreted as a form of self-restitution, in which doing and being were intertwined to restore meaning to life.

The work role loss highlighted the fragility of identity accompanying mental illness. Work served as both a source of income and a way to feel connected to the world. Its absence was experienced as a loss of recognition, but small domestic and community achievements emerged as forms of symbolic resistance, reaffirming the ability to do and contribute. These experiences should not be interpreted as minor adaptations, but as significant expressions of agency and resilience underpinning personal meaning reconstruction. From a clinical practice perspective, this finding reinforces the need to recognize everyday occupations as therapeutic spaces where people regain both identity and confidence.

The family role and the relational environment showed that recovery is not an individual process, but an interdependent one. The balance between support and autonomy proved crucial for family relationships to act as facilitators rather than sources of dependence. Experiences of stigmatization and social exclusion, meanwhile, reflected a profound alteration in the way of being-with-others. Exclusion was not only material or functional, but existential, affecting their sense of belonging. Consequently, community inclusion requires both access to spaces and genuine opportunities for reciprocity and social recognition.

Personal goals, both concrete and symbolic, were interpreted as horizons of meaning that linked the present with the hope of a possible future. The ability to project oneself was configured as an act of resistance in the face of the limitations imposed by their disease and their context. This finding reaffirms that recovery is inseparable from the desire and possibility of imagining a future, even amidst uncertainty. Promoting this horizon of hope should be a priority in clinical and community care.

From an applied perspective, this study provided a comprehensive understanding of the personal and contextual factors influencing recovery, integrating life history and occupational experience into the analysis. These results support the need to incorporate standardized tools into clinical practice that allow for rigorous exploration of individuals’ life history, identity and context. Having validated sensitive instruments for subjectivity, applied by professionals trained in the interpretation of occupation and personal history, can improve the accuracy of assessments and the planning of tailored support. This approach, although interdisciplinary, reinforces the role of occupational analysis to understanding experiences’ meaning and guiding person-centered interventions.

Clinical implications of these findings point to the need to design more personalized community interventions promoting participation in meaningful activities, strengthening feelings of self-efficacy and fostering social integration. The integration of recovery-oriented activities within community settings, such as libraries, civic centers, and other local organizations and the facilitation of group-based activities such as including art therapy, music therapy, theatre, cooking, and sports across these varied community resources can promote the development of new roles and the engagement with novel environments. These initiatives are likely to enhance participation not only within the service but also in the broader community, fostering the formation of new friendships, reducing reliance on institutional settings and ultimately providing individuals with meaningful experiences that contribute to the advancement of their personal life projects.

Additionally, they also address the urgency of strategies to reduce stigma and generate accessible employment and social opportunities. Integrating life history into the assessment and planning of support not only allows for the interventions’ adaptation, but it also dignifies people’s experiences by placing their narrative at the center of the therapeutic process. Furthermore, it is important to state that the application of the OPHI-II in mental health contexts requires sufficient time both for proper administration and for the development of a trusting rapport with participants.

Overall, the findings of this study show that recovery in mental health cannot be understood as a linear or exclusively individual process, but rather as an interdependent experience that is deeply embedded in individuals’ life histories and social contexts. Beyond symptoms or diagnosis, recovery must involve rebuilding bonds, restoring identity and projecting oneself toward a possible future. Incorporating this understanding into interdisciplinary practice can contribute to transforming mental health care models, promoting truly person-centered care based on life history, participation and social inclusion.

## 5. Conclusions

This qualitative study provided an in-depth understanding of how life history, identity, competence and context influence the recovery processes of people with mental illness. The findings indicate that recovery is a dynamic and non-linear process shaped by the meanings individuals assign to their routines, roles, support systems and personal goals.

Beyond symptomatic remission, recovery involved rebuilding identity, reestablishing meaningful connections and finding a sense of purpose in everyday life. The narratives collected show that domestic and community activities, although simple, become spaces for personal and social reaffirmation. Likewise, the need to balance family support with individual autonomy and to generate community opportunities promoting inclusion and social participation was highlighted.

These results reinforce the importance of integrating life history and occupational perspectives into the assessment and planning of personalized supports, moving beyond models focused solely on diagnosis. The systematic incorporation of tools such as OPHI-II can contribute to the design of more sensitive interventions to subjective experience and promote genuinely person-centered care.

## 6. Limitations and Future Research

Among the limitations of this study is the fact that fieldwork was conducted in a single community association, which could restrict the diversity of experience collected and limit the contextual variability of the findings. In addition, the possible influence of gender on recovery experiences could not be explored in depth, as the sample was composed mainly of men. Future studies should consider a more balanced representation that allows for the analysis of possible gender differences.

The decision not to record the interviews, taken to preserve participants’ confidence and comfort, may have reduced the narratives’ textual richness. However, this aspect was mitigated by researcher triangulation, cross-checking and data collective discussion. In a similar manner, the sample’s specific sociocultural context may condition the results’ transferability to other settings.

Looking ahead, it would be relevant to explore in greater depth how biographical and occupational trajectories influence the construction of identity and life purpose throughout the different phases of the recovery process. It would also be relevant to develop comparative studies between different cultural contexts and care models, as well as to evaluate the impact of occupation-based interventions and biographical planning on quality of life, empowerment and social inclusion.

## Data Availability

The data presented in this study are available on request from the corresponding author due to ethical restrictions related to the protection of participant privacy.

## Abbreviations

The following abbreviations are used in this manuscript:

OPHI-II	Occupational Performance History Interview-II
OSF	Open Science Framework
MOHO	Human Occupation Model
COREQ	Consolidated Criteria for Reporting Qualitative Research
